# Dapsone induced hemolysis in a patient with ANCA associated glomerulonephritis and normal G6PD level and implications for clinical practice: case report and review of the literature

**DOI:** 10.1186/s40064-015-0816-y

**Published:** 2015-01-23

**Authors:** Scott M Lee, Duvuru Geetha

**Affiliations:** Division of Nephrology, Johns Hopkins University, 301 Mason Lord Drive, Baltimore, MD 21224 USA

## Abstract

Dapsone is a commonly used second line drug for prophylaxis of pneumocystis jirovecii pneumonia (PJP) in immunocompromised patients. Oxidant hemolysis, caused by dapsone’s metabolite hydroxylamine, is a common side effect, and screening for glucose-6-phosphate dehydrogenase (G6PD) deficiency is recommended before the drug is started in order to prevent potential hemolytic reactions. We report a case of dapsone induced hemolytic anemia in a patient with ANCA associated glomerulonephritis and normal G6PD level. Her anemia improved after cessation of therapy with dapsone. We review the literature of dapsone induced hemolysis in patients who have normal G6PD level and discuss potential pathways leading to hemolytic anemia and its implications for clinical practice.

## Introduction

Pneumocystis jirovecii pneumonia (PJP) is a common infectious complication in immunocompromised patients including HIV patients and organ transplant recipients. Immunosuppressive drug treatment is used to induce remission in patients with ANCA associated vasculitis (AAV). The ANCA associated vasculitides include granulomatosis with polyangiitis, microscopic polyangiitis and eosinophilic granulomatosis with polyangiitis. The reported rate of PJP in immunocompromised AAV patients ranges from 1% to 20% (Guillevin et al. 1997; Ognibene et al. 1995; Reinhold-Keller et al. 2000). Although there are no randomized controlled trials, data from case series and observational data from trials support the use of PJP prophylaxis due to high mortality associated with PJP. The current European League against Rheumatism (EULAR) recommends the use of PJP prophylaxis for the management of vasculitis (Mukhtyar et al. 2009).

Dapsone has been shown to be effective for PJP prophylaxis for HIV patients in at least 41 clinical trials of HIV patients (Hughes 1998). Dapsone is a commonly used alternative for prophylaxis of pneumocystis jerovicci pneumonia (PJP) in immunocompromised patients who are intolerant to trimethoprim and sulfamethoxazole (Hughes 1998). Oxidant hemolysis caused by its metabolite hydroxylamine is a reported side effect in patients with glucose-6-phosphate dehydrogenase (G6PD) deficiency and therefore screening for G6PD deficiency is recommended before the drug is started. Although dapsone induced hemolytic anemia is mostly reported in patients with G6PD deficiency, there are reports of dapsone induced hemolytic anemia in literature regarding transplant recipients who have normal G6PD levels (Lee et al. 2005; Naik et al. 2008; Olteanu et al. 2012). Dapsone is also often used for PJP prophylaxis in patients with AAV who receive immunosuppressive drug therapy to induce remission. We report the first case of dapsone induced hemolytic anemia in a patient with AAV in the setting of a normal G6PD level. We provide a literature review of dapsone induced hemolysis in patients who have normal G6PD level and discuss potential pathways leading to hemolytic anemia in this setting and its implications for patient care.

## Case report

A 60 year old Caucasian female was diagnosed with ANCA associated vasculitis in October, 2013 when she presented acute renal failure with a serum creatinine of 2.4 mg/dl, hematuria and proteinuria with MPO ANCA positivity, and a renal biopsy revealing pauci-immune necrotizing and crescentic glomerulonephritis. She was initially treated with oral cyclophosphamide and prednisone and after one month, her cyclophosphamide was stopped and she was given rituximab for remission induction. Due to her renal insufficiency, she was started on dapsone for pneumocystis jirovecii pneumonia prophylaxis after confirmation of normal glucose-6-phosphate dehydrogenase (G6PD) activity level by quantitative testing twice. Her serum creatinine peaked at 3.1 mg/dl and improved to 2.16 mg/dl with immunosuppressive therapy. Her hemoglobin prior to initiation of dapsone was 11.1. About a month later, her hemoglobin was noted on routine labs to be 7.3 with an elevated MCV of 104 and elevated corrected reticulocyte count of 11.3%. She had normal iron stores, serum B12 and folate levels. Her haptoglobulin was less than 7. Her dapsone was stopped with improvement in her hemoglobin to 8.1, five days post discontinuation of dapsone therapy.

## Discussion

We report the first case of dapsone induced hemolysis in the setting of a normal G6PD level in a patient with ANCA associated vasculitis which improved after dapsone was discontinued. We speculate that the severe renal dysfunction in our patient could have led to high dapsone blood level predisposing to hemolysis despite the presence of normal G6PD activity level. This case highlights the need for close clinical follow up in high risk patients.

G6PD deficiency is one of the most common genetic disorders of red blood cells worldwide with an estimated 400 million people carrying a mutation of the gene that causes G6PD deficiency. The red blood cells depend upon an adequate supply of reduced glutathione which helps to detoxify hydrogen peroxide and other reactive oxygen species. The role of G6PD in red blood cells is to provide NADPH and maintain an adequate supply of reduced glutathione (Mason et al. 2007). When there is a deficiency of G6PD and red blood cells are exposed to oxidative challenge such as exposure to medications like dapsone, the glutathione stores are rapidly exhausted and as a result reactive oxygen species are not detoxified (Beutler et al. 1957). This results in attack of sulfhydryl groups in hemoglobin which causes damage to the cell membrane, formation of Heinz bodies, and eventually the destruction of red cells (Fischer et al. 1985; Luzzatto and Seneca 2014).

Dapsone induced hemolysis occurs in approximately 4% of HIV patients receiving dapsone for PJP prophylaxis (Hughes 1998). In the setting of solid organ transplant, the incidence of dapsone induced hemolytic anemia in patients with normal G6PD level ranges from 23 % to 76% (Lee et al. 2005; Naik et al. 2008). In SCT recipients, the incidence of hemolysis is 87% (Olteanu et al. 2012). Anemia caused by dapsone induced hemolysis can be mild and asymptomatic or severe requiring blood transfusion. We summarize the data from these case series in Table 1.

**Table 1:** **Summary of studies of dapsone induced hemolytic anemia with normal G6PD activity**

**Ref**	**Number of subjects**	**Clinical setting**	**Dapsone dose**	**G6PD activity tested (n)**	**Median days to hemolysis (range)**	**Need for blood transfusion (n)**
6	11	Solid organ transplant	100 mg	6	23 (7–735)	4
7	10	Lung transplant	100 mg	9	83 (46–156)	7
8	26	Stem cell transplant	100 mg	26	13(7–36)	NA

Lee et al. reported 11 cases of dapsone induced hemolytic anemia in recipients of solid organ transplants (3 kidney, 2 liver, 2 heart, 1 lung, 1 kidney and liver, 1 heart and kidney, 1 heart and lung) (Lee et al. 2005). There were 6 male patients and 9 were Caucasian. Hemolysis was defined as a decrease in hemoglobin with presence of at least one laboratory marker of hemolysis and an improvement to baseline hemoglobin after discontinuation of dapsone. Only six patients had G6PD level checked prior to treatment and all six patients had normal levels. The mean decrease in hemoglobin was 2.5 g/dL. Four patients required transfusion of packed red blood cells. The majority of these patients were on tacrolimus based immunosuppression. It was postulated that elevated dapsone blood levels caused by concurrent therapy with tacrolimus due to sharing of the cytochrome P-450 isoenzyme for metabolism by these drugs may have predisposed to hemolysis.

A retrospective review of 43 lung transplant recipients by Naik et al. identified ten patients with dapsone induced hemolytic anemia defined as low serum haptoglobulin with simultaneous decrease in hemoglobin (Naik et al. 2008). Of the 10 patients, 9 were tested for G6PD activity and had normal G6PD level. Nine patients were Caucasian and 7 were females. Mean drop in hemoglobin was 2.7 g/dL. Peak serum creatinine ranged from 0.8 to 4.1 mg/dl. Patients with increased serum creatinine and lower body weight were at higher risk of hemolysis suggesting that dose adjustment for dapsone may be needed in patients with renal insufficiency and lower body weight.

Another retrospective study of 30 recipients with stem cell transplants and normal G6PD level treated with dapsone for PJP prophylaxis identified 26 cases of dapsone induced hemolysis (Olteanu et al. 2012). In this series, the hemolysis, defined by a peripheral blood smear evidence of bite cells and eccentrocytes, was mild and patients were continued on dapsone with no adverse consequences.

The occurrence of hemolysis in patients with normal G6PD level suggest that the hemolysis may be a dose related event as may be seen with elevated dapsone levels in patients with renal dysfunction or due to concurrent use of medications that use the cytochrome P-450 isoenzyme system. Alternatively, there may be mutations in hexose monophosphate shunt or glutathione metabolism that lead to glutathione depletion and subsequent hemolysis.

## Conclusion

In patients with renal dysfunction, lower body weight or use of concurrent medications that interact with dapsone, we propose that regular monitoring of hemoglobin should be done after initiation of dapsone even if G6PD level is normal.
